# Supplementary material for: Evaluation of vectors for gene expression in Pseudovibrio marine bacteria
Source: Appl Environ Microbiol. 2025 Mar 4;91(4):e00207-25. doi: 10.1128/aem.00207-25 (PMC12016493; doi:10.1128/aem.00207-25)
Supplement: Supplemental material — Tables S1 to S6 and Figures S1 to S6. [file aem.00207-25-s0001.pdf]

Supplementary Information

Evaluation of vectors for gene expression in *Pseudovibrio* marine bacteria

Yitao Dai,[1] Alessandra S. Eustáquio[1*]

[1]Department of Pharmaceutical Sciences and Center for Biomolecular Sciences, College of Pharmacy, University of Illinois at Chicago, Chicago, IL, USA

Table of Contents

Table S1. Illumina sequencing results of *P. brasiliensis* Ab134 carrying different plasmids. **3**

Table S2. Area under the curve and lag phase of *P. brasiliensis* Ab134 carrying different plasmids. **4**

Table S3. Electroporation efficiency of pAM4891 and pSEVA237R_Pem7 into *P. brasiliensis* Ab134. .... **5**

Table S4. Conjugation efficiency of pAM4891 and pSEVA237R_Pem7 into *P. brasiliensis* Ab134 from *E. coli* S17-1. .... **6**

Table S5. Plasmid stability of pAM4891 and pSEVA237R_Pem7 in *P. brasiliensis* Ab134. .... **7**

Table S6. Concentration of plasmid pAM4891 and pSEVA237R_Pem7 measured by NanoDrop. **8**

Figure S1. Plasmid vector DNA isolated from *P. brasiliensis* Ab134. .... **9**

Figure S2. Growth of independent clones of *P. brasiliensis* Ab134 containing the pBBR1 replicon vectors. **10**

Figure S3. Filter mating plates for evaluating conjugation efficiency.. .... **12**

Figure S4. Fluorescence imaging of quadruplicate images of electroporation results. **13**

Figure S5. Fluorescence imaging of quadruplicate plasmid stability results. **14**

Figure S6. Triplicate swarming motility assays of *P. brasiliensis* Ab134 wild type or *∆pppA* mutant compared to strains carrying self-replicative vectors. **15**

Tables

Table S1. Illumina sequencing results of *P. brasiliensis* Ab134 carrying different plasmids.

**Plasmid vector name**	**pAM4891**	**pSEVA234M**	**pSEVA237R_Pem7**
**Replicon**	RSF1010	pBBR1	pBBR1
**Total Reads**	5,029,942	3,427,816	4,393,640
**Assembled to Reference (%)**	98.95%	97.75%	99.33%
**Chromosome Coverage**	111.9	66.2	94.4
**Plasmid 1 Coverage**	122.8	74.8	103
**Plasmid 2 Coverage**	117.7	82.1	98.2
**Plasmid 3 Coverage**	170	124.2	160.2
**Plasmid 4 Coverage**	155.8	115.8	142.9
**Plasmid 5 Coverage**	109.3	94.9	116
**Conjugated Plasmid Coverage**	5,525.6	13,838.3	13,957.5

Table S2. Area under the curve and lag phase of *P. brasiliensis* Ab134 carrying different plasmids.

Strains	Area under the curve (AUC)	Lag phase (h)
WT_1	37.21	2.5
WT_2	35.52	2.5
WT_3	39.51	2.5
WT_4	38.16	2.5
WT_5	44.69	2.5
WT_6	36.24	2.5
WT_7	34.11	2.5
WT_8	34.84	2.5
WT_9	34.10	2.5
WT_10	34.40	2.5
pAM4891_1	35.12	3.5
pAM4891_2	33.76	3.5
pAM4891_3	33.64	3.5
pAM4891_4	34.22	3.5
pAM4891_5	32.45	3.5
pAM4891_6	33.50	3.5
pAM4891_7	32.77	3.5
pAM4891_8	32.97	3.5
pAM4891_9	32.02	3.5
pAM4891_10	32.68	3.5
pSEVA234M_1	16.36	2
pSEVA234M_2	16.00	2.5
pSEVA234M_3	15.99	2.5
pSEVA234M_4	16.40	2.5
pSEVA234M_5	16.50	2.5
pSEVA234M_6	15.71	2.5
pSEVA234M_7	16.44	2.5
pSEVA234M_8	16.25	2.5
pSEVA234M_9	16.15	2.5
pSEVA234M_10	15.87	2.5
pSEVA237R_Pem7_1	41.08	2.5
pSEVA237R_Pem7_2	41.00	2.5
pSEVA237R_Pem7_3	40.98	2.5
pSEVA237R_Pem7_4	40.50	2.5
pSEVA237R_Pem7_5	39.62	2.5
pSEVA237R_Pem7_6	40.34	3.0
pSEVA237R_Pem7_7	40.56	3.0
pSEVA237R_Pem7_8	42.18	3.0
pSEVA237R_Pem7_9	42.13	3.0
pSEVA237R_Pem7_10	40.33	3.0

Table S3. Electroporation efficiency of pAM4891 and pSEVA237R_Pem7 into *P. brasiliensis* Ab134.

Plasmid	pAM4891	pSEVA237R_Pem7
Fluorescent CFU (triplicates)	383	412
	451	306
	412	434
Average CFU	415	384
Amount of plasmid DNA (µg)	0.126	0.138
CFU per µg plasmid DNA	3,295 ± 271	2,776 ± 495

Table S4. Conjugation efficiency of pAM4891 and pSEVA237R_Pem7 into *P. brasiliensis* Ab134 from *E. coli* S17-1.

Plasmid	CFU×mL^{-1} (triplicates)	Dilution factor	Conjugation efficiency
pAM4891	$1.4×10^5$	10^4	$3.86×10^{-6} ± 3.34×10^{-7}$
	$1.2×10^5$		
	$1.4×10^5$		
Average CFU×mL^{-1}	$1.3×10^5 ± 1.2×10^4$		
pSEVA237R_Pem7	$5.0×10^7$	10^6	$2.22×10^{-3} ± 6.69×10^{-4}$
	$9.0×10^7$		
	$9.0×10^7$		
Average CFU×mL^{-1}	$7.7×10^7 ± 2.3×10^7$		
Total recipient CFU×mL^{-1} *P. brasiliensis* Ab134	$3.38×10^{10}$	10^8	---
	$3.54×10^{10}$		
	$3.44×10^{10}$		
Average CFU×mL^{-1}	$3.45×10^{10} ± 8.08×10^8$		

Table S5. Plasmid stability of pAM4891 and pSEVA237R_Pem7 in *P. brasiliensis* Ab134.

Time (h)	Plasmid	Type	CFU×mL^{-1} (quadruplicates)				Average ratio	Dilution factor
T0	pAM4891	Glowing	1.12×10^{10}	1.00×10^{10}	8.7×10^{9}	8.9×10^{9}	100%	10^{8}
		Total	1.12×10^{10}	1.00×10^{10}	8.7×10^{9}	8.9×10^{9}		
		Ratio	100%	100%	100%	100%		
	pSEVA237R_Pem7	Glowing	1.62×10^{10}	1.28×10^{10}	1.20×10^{10}	1.40×10^{10}	100%	
		Total	1.62×10^{10}	1.28×10^{10}	1.20×10^{10}	1.40×10^{10}		
		Ratio	100%	100%	100%	100%		
T24	pAM4891	Glowing	1.07×10^{10}	1.55×10^{10}	1.55×10^{10}	1.24×10^{10}	99.38%	10^{8}
		Total	1.09×10^{10}	1.55×10^{10}	1.56×10^{10}	1.24×10^{10}		
		Ratio	98.17%	100%	99.36%	100%		
	pSEVA237R_Pem7	Glowing	1.91×10^{10}	2.19×10^{10}	1.82×10^{10}	1.92×10^{10}	100%	
		Total	1.91×10^{10}	2.19×10^{10}	1.82×10^{10}	1.92×10^{10}		
		Ratio	100%	100%	100%	100%		
T48	pAM4891	Glowing	1.77×10^{10}	1.26×10^{10}	1.33×10^{10}	1.29×10^{10}	99.62%	10^{8}
		Total	1.77×10^{10}	1.26×10^{10}	1.33×10^{10}	1.31×10^{10}		
		Ratio	100%	100%	100%	98.47%		
	pSEVA237R_Pem7	Glowing	2.26×10^{10}	1.77×10^{10}	2.42×10^{10}	2.41×10^{10}	100%	
		Total	2.26×10^{10}	1.77×10^{10}	2.42×10^{10}	2.41×10^{10}		
		Ratio	100%	100%	100%	100%		
T72	pAM4891	Glowing	1.06×10^{10}	1.38×10^{10}	1.41×10^{10}	1.53×10^{10}	97.25%	10^{8}
		Total	1.10×10^{10}	1.40×10^{10}	1.45×10^{10}	1.58×10^{10}		
		Ratio	96.36%	98.57%	97.24%	96.84%		
	pSEVA237R_Pem7	Glowing	2.94×10^{10}	2.64×10^{10}	3.27×10^{10}	2.93×10^{10}	100%	
		Total	2.94×10^{10}	2.64×10^{10}	3.27×10^{10}	2.93×10^{10}		
		Ratio	100%	100%	100%	100%		
T96	pAM4891	Glowing	1.78×10^{10}	1.40×10^{10}	1.20×10^{10}	1.33×10^{10}	85.44%	10^{8}
		Total	2.03×10^{10}	1.59×10^{10}	1.36×10^{10}	1.71×10^{10}		
		Ratio	87.68%	88.05%	88.24%	77.78%		
	pSEVA237R_Pem7	Glowing	1.8×10^{10}	2.2×10^{10}	1.7×10^{10}	1.7×10^{10}	100%	10^{9}
		Total	1.8×10^{10}	2.2×10^{10}	1.7×10^{10}	1.7×10^{10}		
		Ratio	100%	100%	100%	100%		
T120	pAM4891	Glowing	1.40×10^{10}	1.44×10^{10}	1.12×10^{10}	1.12×10^{10}	76.6%	2×10^{8}
		Total	1.72×10^{10}	1.92×10^{10}	1.12×10^{10}	1.12×10^{10}		
		Ratio	81.4%	75%	65.06%	84.93%		
	pSEVA237R_Pem7	Glowing	2.66×10^{10}	1.92×10^{10}	2.50×10^{10}	1.90×10^{10}	100%	
		Total	2.66×10^{10}	1.92×10^{10}	2.50×10^{10}	1.90×10^{10}		
		Ratio	100%	100%	100%	100%		

Table S6. Concentration of plasmid pAM4891 and pSEVA237R_Pem7 measured by NanoDrop.

Plasmid	Concentration (ng/μL)	Average concentration (ng/μL)	260 nm/280 nm	260 nm/230 nm
pAM4891	126.3	126.0	1.89	2.17
	126.8		1.89	2.16
	125.0		1.99	2.15
pSEVA237R_Pem7	70.4	69.2	1.92	2.10
	69.3		1.98	2.13
	67.8		1.86	1.93

Figures

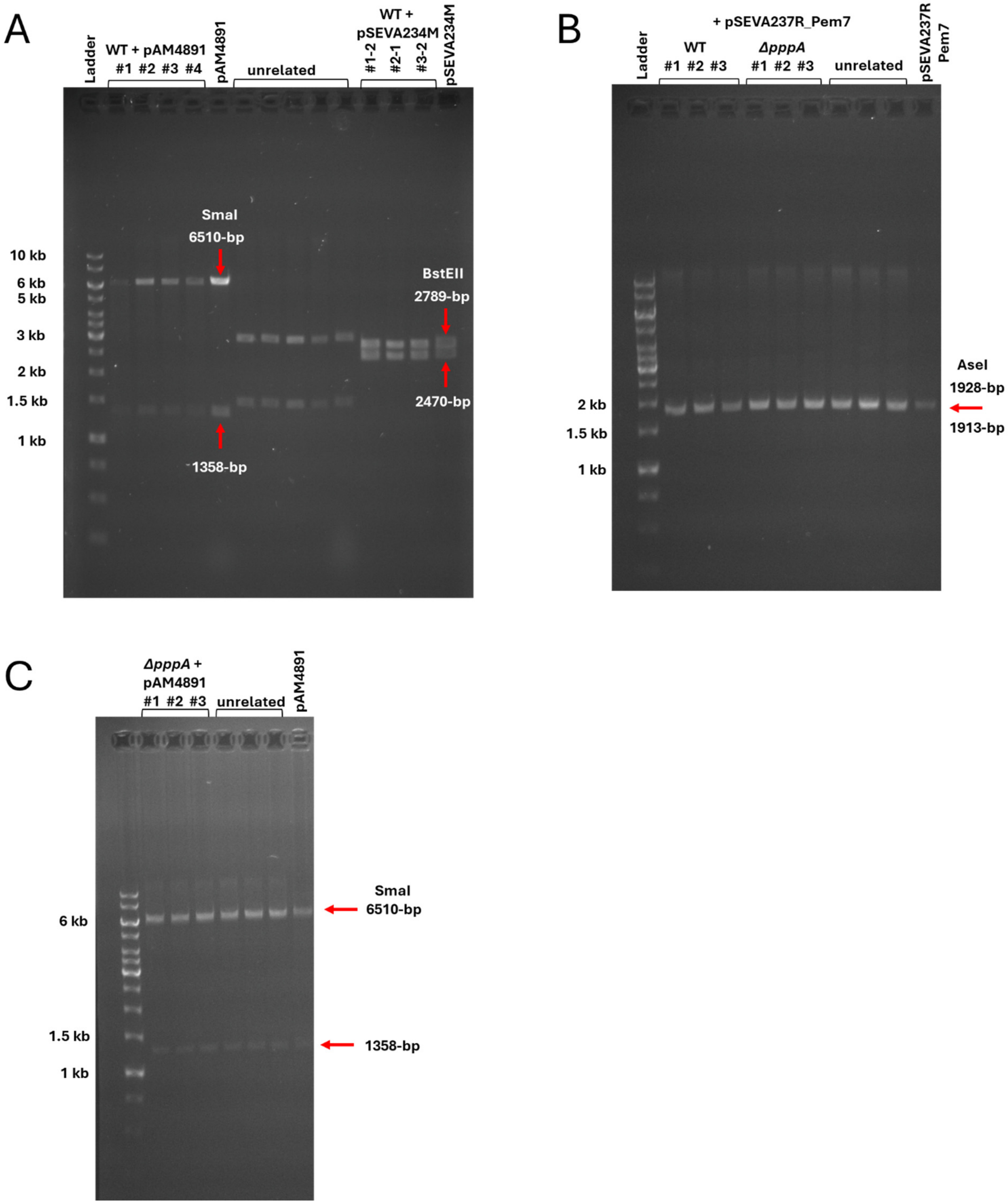

Figure S1. Plasmid vector DNA isolated from *P. brasiliensis* Ab134. (**A**) Gel electrophoresis analysis of pAM4891 (RSF1010 replicon) and pSEVA234M (pBBR1) isolated from *P. brasiliensis* Ab134 wild type and digested with SmaI and BstEII respectively. (**B**) Gel electrophoresis analysis of pSEVA237R_Pem7 isolated from *P. brasiliensis* Ab134 wild type and Δ*pppA* mutants. AseI was

used for digestion. (**C**) Gel electrophoresis analysis of pAM4891 isolated from *P. brasiliensis* Δ*pppA* mutants. SmaI was used for digestion.

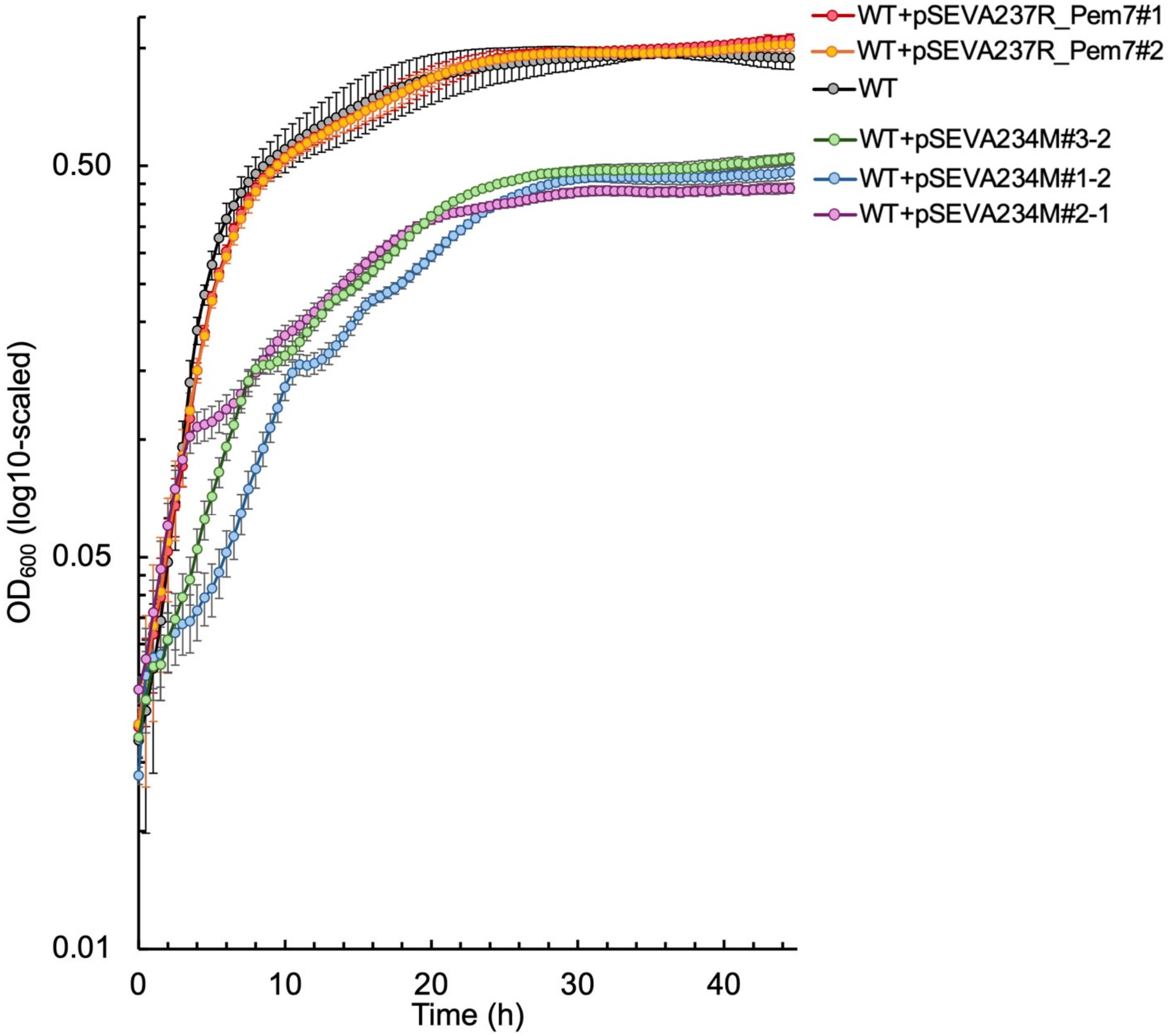

Figure S2. Growth of independent clones of *P. brasiliensis* Ab134 containing the pBBR1 replicon vectors. Growth in liquid cultures was measured by OD_{600}. *N*=10. Error bars indicate standard deviation.

A

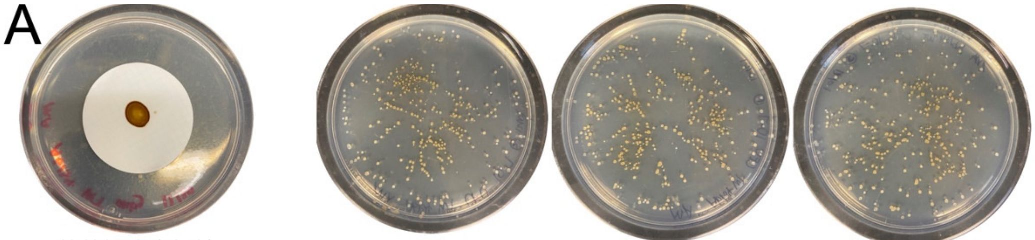

Ab134 only (50 µL)

10^{-8} dilution for CFU counting (triplicates)

B

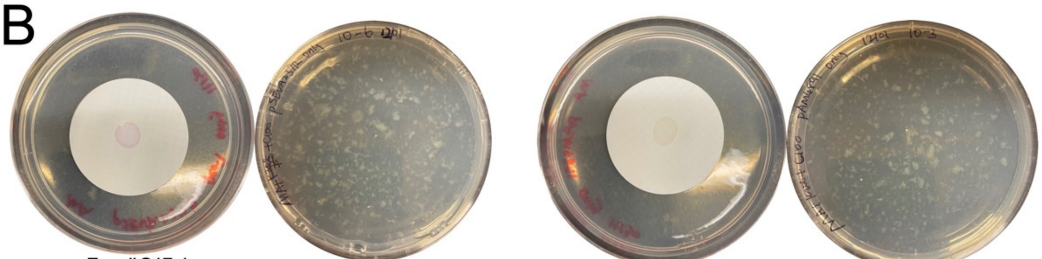

E. coli S17-1
pSEVA237R_Pem7 only (50 µL)

10^{-7} dilution (control)

E. coli S17-1
pAM4891 only (50 µL)

10^{-4} dilution (control)

C

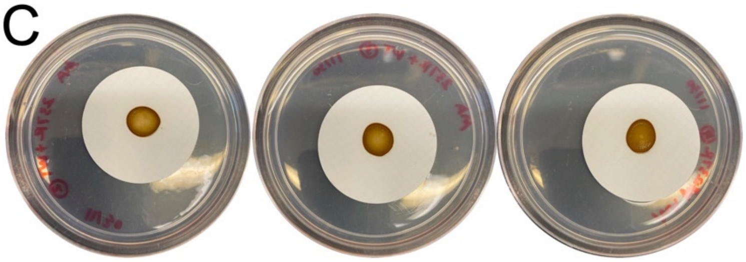

Ab134 (50 µL) + pSEVA237R_Pem7 (50 µL)

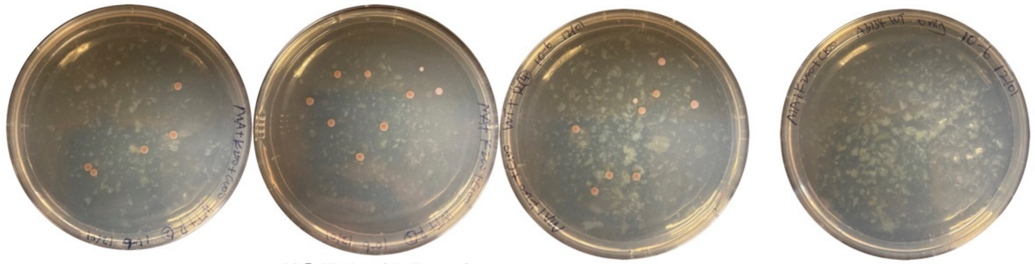

10^{-7} dilution (triplicates)

Ab134 only
10^{-7} dilution (control)

D

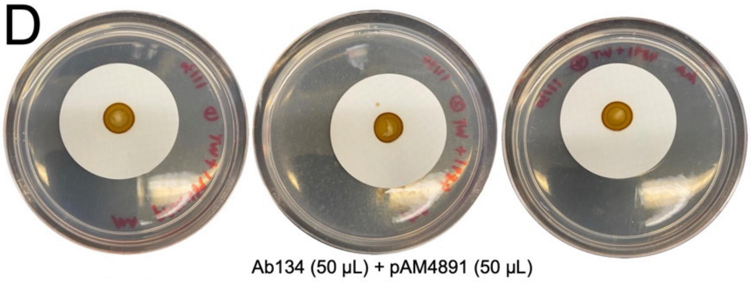

Ab134 (50 µL) + pAM4891 (50 µL)

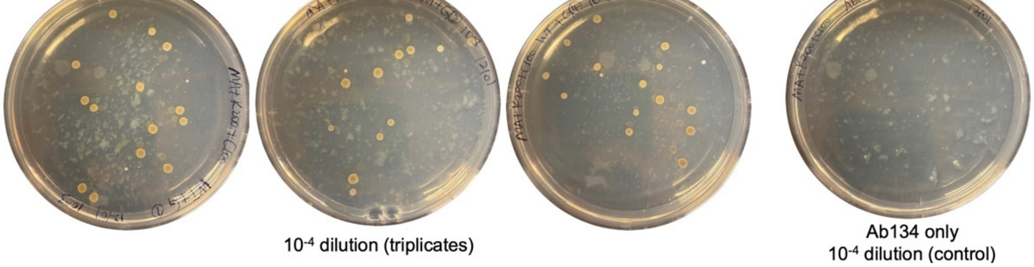

10^{-4} dilution (triplicates)

Ab134 only
10^{-4} dilution (control)

Figure S3. Filter mating plates for evaluating conjugation efficiency. (**A**) *Pseudovibrio brasiliensis* Ab134 control on filter paper (left) and triplicate 10^{-8} dilutions on marine agar plates without antibiotics for $CFU \cdot mL^{-1}$ counting. (**B**) *Escherichia coli* S17-1 controls carrying either pSEVA237R_Pem7 or pAM4891, with corresponding dilutions on marine agar plates containing kanamycin (200 µg/mL) and carbenicillin (50 µg/mL). (**C**) Conjugation mixtures of *P. brasiliensis* Ab134 and *E. coli* S17-1 carrying pSEVA237R_Pem7 in triplicates, alongside dilutions on marine agar plates containing antibiotics. The 10^{-7} dilution plate of *P. brasiliensis* Ab134 only is shown as a control. (**D**) Conjugation mixtures of *P. brasiliensis* Ab134 and *E. coli* S17-1 carrying pAM4891 in triplicates, alongside dilutions on marine agar plates containing antibiotics. The 10^{-4} dilution of *P. brasiliensis* Ab134 only is shown as a negative control.

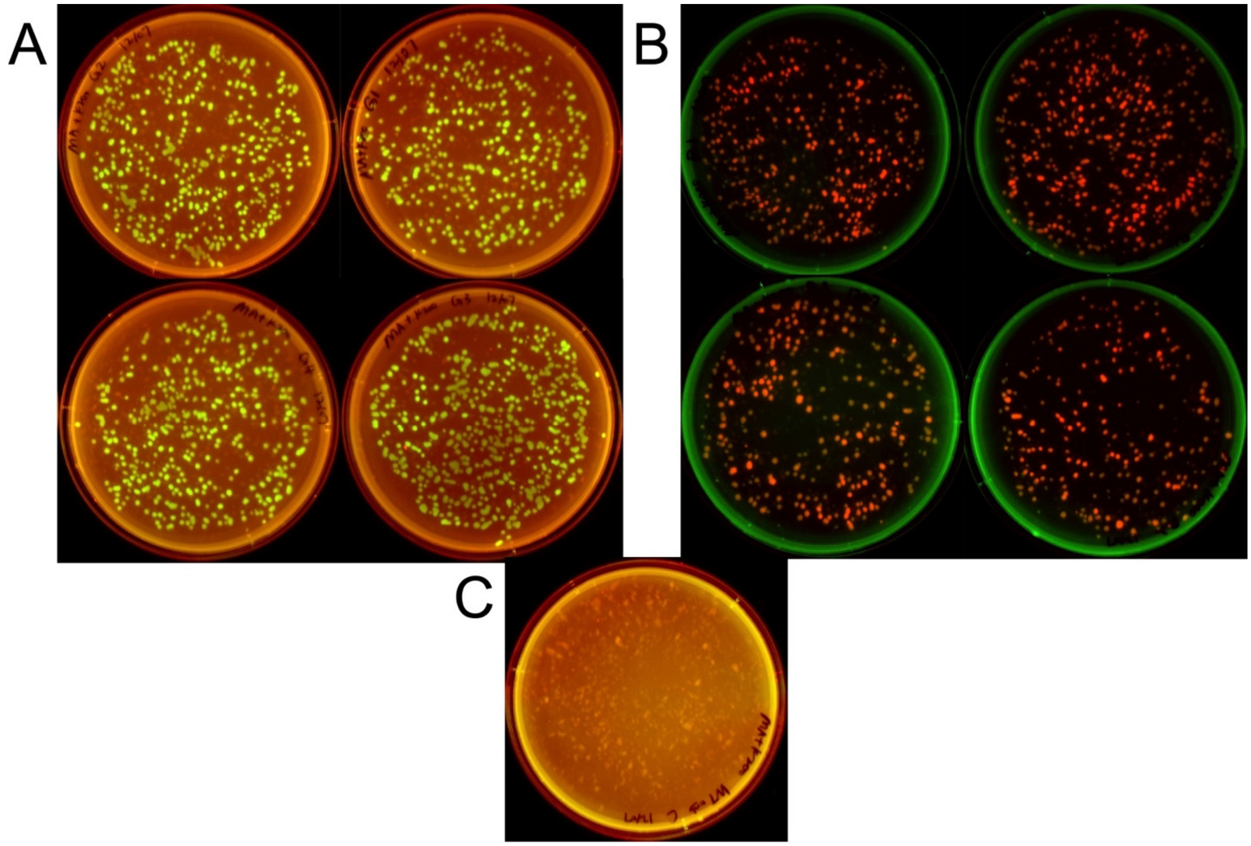

Figure S4. Fluorescence imaging of quadruplicate images of electroporation results. (**A**) pAM4891, green fluorescence. (**B**) pSEVA237R_Pem7, red fluorescence. (**C**) Control with no plasmid electroporated. Multi-channel (auto-exposure) with Cy2 (532 nm / 28 mm) and Alexa 546 (602 nm / 50 mm) used to visualize green and red fluorescence, respectively.

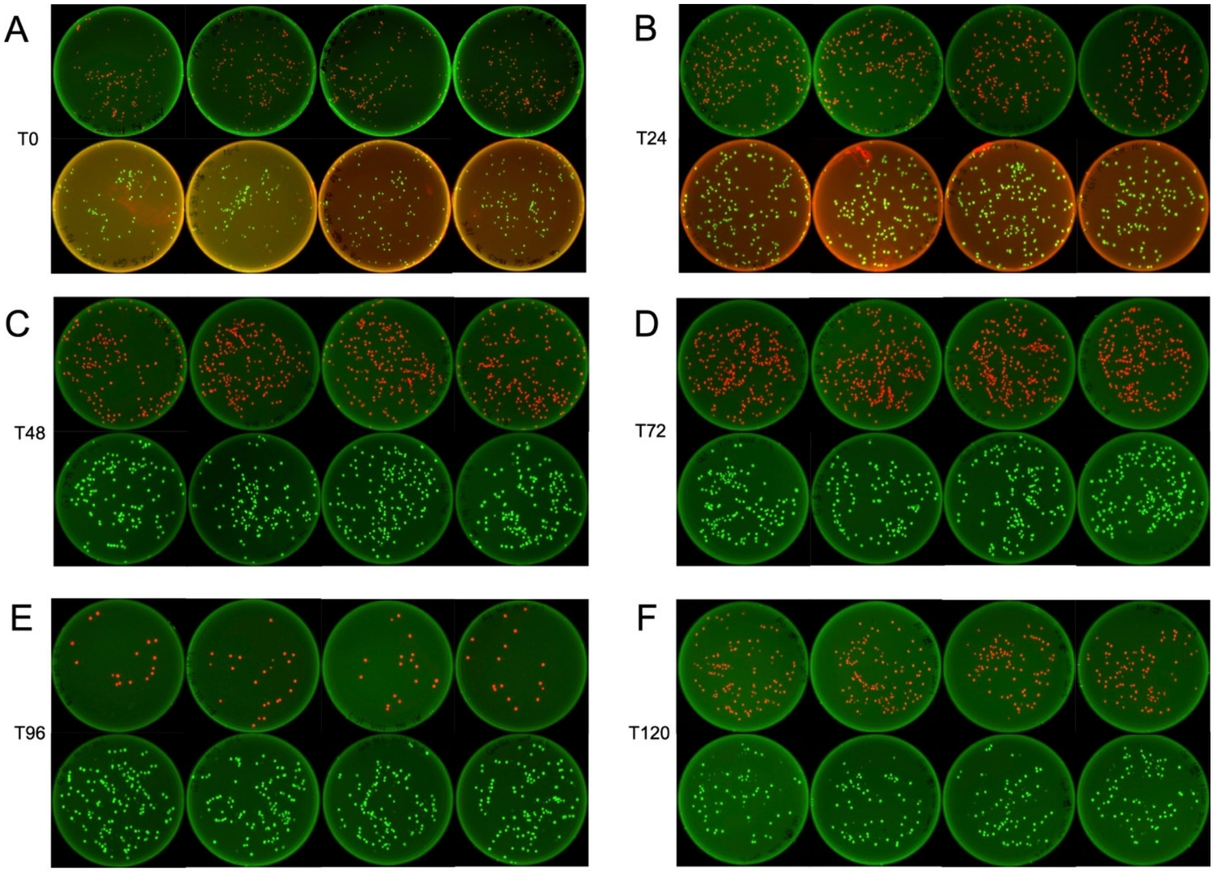

Figure S5. Fluorescence imaging of quadruplicate plasmid stability results. Dilution factors are listed in **Table S5**. (**A**) Initial seed culture at T0. (**B**) 1st passage at T24. (**C**) 2nd passage at T48. (**D**) 3rd passage at T72. (**E**) 4th passage at T96. (**F**) 5th passage at T120. pAM4891, green fluorescence; pSEVA237R_Pem7, red fluorescence. Multi-channel (auto-exposure) with Cy2 (532 nm / 28 mm) and Alexa 546 (602 nm / 50 mm) used to visualize green and red fluorescence, respectively.

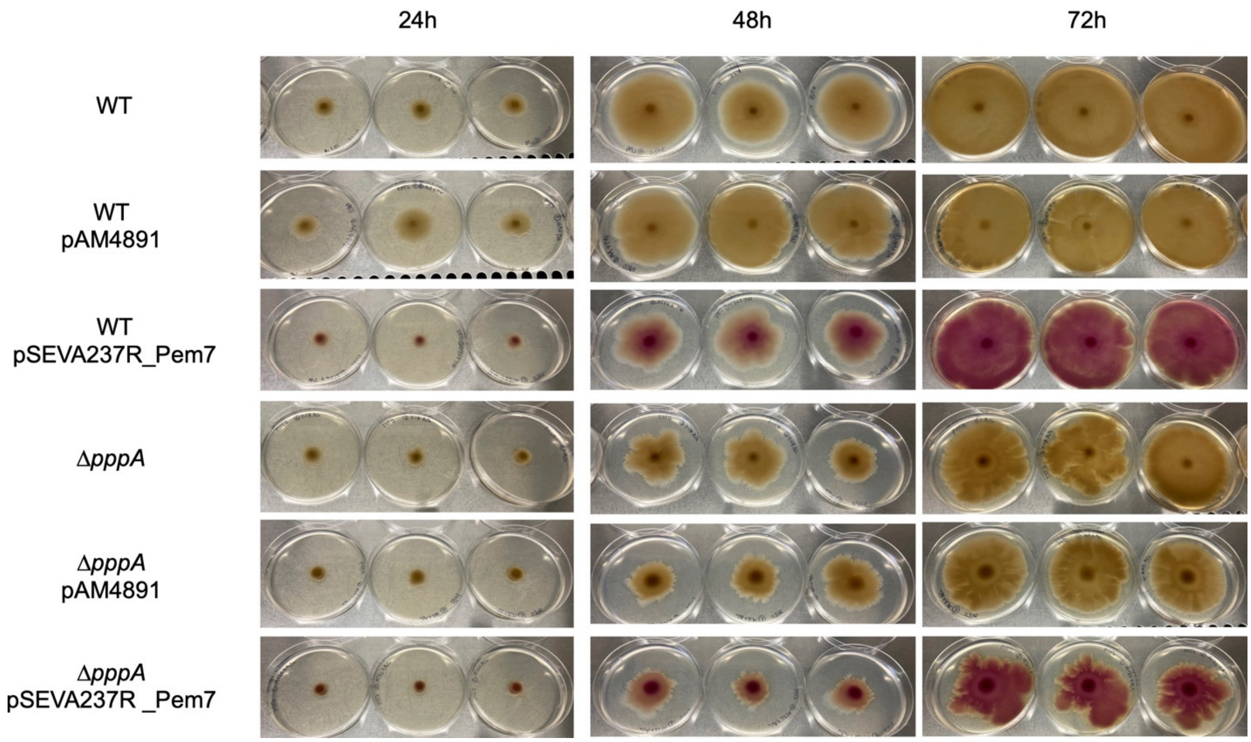

Figure S6. Triplicate swarming motility assays of *P. brasiliensis* Ab134 wild type or *ΔpppA* mutant compared to strains carrying self-replicative vectors. Swarming assays performed on marine broth with 0.5 % Eiken agar. Pictures shown were taken at 24, 48, and 72 hours after inoculation, respectively.
